# Carotid artery ligation induced intimal thickening and proliferation is unaffected by ageing

**DOI:** 10.1007/s12079-017-0431-5

**Published:** 2017-11-29

**Authors:** B. A. Brown, H. Williams, A. R. Bond, G. D. Angelini, J. L. Johnson, S. J. George

**Affiliations:** Bristol Medical School, , University of Bristol, Research Floor Level Seven, Bristol Royal Infirmary, Upper Maudlin Street, Bristol, BS2 8HW UK

**Keywords:** Cardiovascular, Neointima, Proliferation, Restenosis, Vascular smooth muscle cell, Wnt

## Abstract

Following interventions to treat atherosclerosis, such as coronary artery bypass graft surgery, restenosis occurs in approximately 40% of patients. Identification of proteins regulating intimal thickening could represent targets to prevent restenosis. Our group previously demonstrated that in a murine model of vascular occlusion, Wnt4 protein expression and β-catenin signalling was upregulated which promoted vascular smooth muscle cell (VSMC) proliferation and intimal thickening. In this study, the effect of age on VSMC proliferation, intimal hyperplasia and Wnt4 expression was investigated. In vitro proliferation of VSMCs isolated from young (2 month) or old (18–20 month) C57BL6/J mice was assessed by immunocytochemistry for EdU incorporation. As previously reported, 400 ng/mL recombinant Wnt4 protein increased proliferation of VSMCs from young mice. However, this response was absent in VSMCs from old mice. As our group previously reported reduced intimal hyperplasia in Wnt4^+/−^ mice compared to wildtype controls, we hypothesised that impaired Wnt4 signalling with age may result in reduced neointimal formation. To investigate this, carotid artery ligation was performed in young and old mice and neointimal area was assessed 21 days later. Surprisingly, neointimal area and percentage lumen occlusion were not significantly affected by age. Furthermore, neointimal cell density and proliferation were also unchanged. These data suggest that although Wnt4-mediated proliferation was impaired with age in primary VSMCs, carotid artery ligation induced neointimal formation and proliferation were unchanged in old mice. These results imply that Wnt4-mediated proliferation is unaffected by age in vivo*,* suggesting that therapeutic Wnt4 inhibition could inhibit restenosis in patients of all ages.

**Electronic supplementary material:**

The online version of this article (10.1007/s12079-017-0431-5) contains supplementary material, which is available to authorized users.

## Introduction

Atherosclerosis is the development and progression of lipid-rich inflammatory plaques within the vascular wall, which culminate in major causes of global mortality such as coronary artery disease and stroke (WHO 2011; McLaren et al. 2011 and Libby 2012). Interventions to treat atherosclerosis, such as balloon angioplasty, intracoronary stent implantation or coronary artery bypass graft surgery, are frequently employed to limit the ischemia caused by this disease. However, in a proportion of patients restenosis of the vessel and re-emergence of ischemic symptoms can occur (reviewed by Schwartz et al. 1995 and Wallitt et al. 2007). In a population of Canadian veterans, 10 year patency (defined as no restenosis) after coronary artery bypass grafting was shown to be 61% in saphenous vein grafts (Goldman et al. 2004). Consequently, 39% of vein grafts were restenotic at this time point, representing a substantial window to improve outcomes following this surgery (Goldman et al. 2004). The need to tackle restenosis is likely to be an increasing problem in the current ageing population (United Nations 2015) as patients would be expected to live for many decades following treatment. That said, exactly how ageing affects restenotic biology has not been fully defined.

An underlying mechanism in restenosis is enhanced vascular smooth muscle cell (VSMC) proliferation and migration leading to the formation of a thickened intima, also termed a neointima, and eventual occlusion of the vessel or graft (reviewed by Schwartz et al. 1995 and Wallitt et al. 2007). Accumulating evidence suggests that following vascular injury, the canonical Wnt signalling pathway is activated, and is at least in part responsible for this enhanced VSMC proliferation and migration (Tsaousi et al. 2011 and Williams et al. 2016a). Activation of the Wnt/β-catenin pathway has been shown to increase VSMC proliferation in vitro (Uglow et al. 2003; Slater et al. 2004; Quasnichka et al. 2006; Tsaousi et al. 2011). In addition, studies have reported increased β-catenin protein (Wang et al. 2002; Slater et al. 2004; Hua et al. 2014; Hua et al. 2015) and induction of β-catenin nuclear translocation (Slater et al. 2004) following balloon injury in rat carotid arteries, while co-localisation of β-catenin/T-cell factor (TCF) signalling and cell proliferation has been described in the neointima of murine carotid arteries following ligation (Tsaousi et al. 2011). Recently we have also demonstrated that targeted suicide of cells with β-catenin/TCF signalling retards intimal thickening, illustrating the importance of this pathway in restenosis (Williams et al. 2016b).

Our group has reported that Wnt4 promotes VSMC proliferation in vitro*,* is upregulated during neointimal formation after carotid artery ligation in mice, and temporally coincided with activation of a β-catenin/TCF responsive reporter (Tsaousi et al. 2011). A causative role for Wnt4 in ligation-induced neointimal thickening was established by demonstrating reduced lesion size, neointimal proliferation and β-catenin nuclear translocation in heterozygous Wnt4 knockout mice compared to wild type controls (Tsaousi et al. 2011). Furthermore, studies by Hua and colleagues found that expression of Wnt4 and β-catenin correlated with induction of neointimal thickening following balloon injury in rat carotid arteries (Hua et al. 2014; Hua et al. 2015). Together these papers suggest that following vascular injury, Wnt4/β-catenin signalling is activated and is at least in part responsible for the induction of VSMC proliferation observed thereafter.

The effect of age on VSMC proliferation is controversial. Although multiple studies in rat VSMCs have reported enhanced proliferation with age, contradictory data has arisen from studies in mouse and human VSMCs (see review by Monk and George 2014). Inevitably, the effect of age on neointima formation is also unclear and reports of both enhanced and reduced neointima formation with age exist. Vazquez-Padron and colleagues reported increased neointimal thickening in wire injured carotid arteries from old mice compared to young controls (Vazquez-Padron et al. 2004). Similar findings have been reported in ageing rats subjected to aortic autografting or wire induced injury (Hariri et al. 1986). However, Torella and co-workers described decreased VSMC proliferation and intimal thickening with age after balloon injury in rat carotid arteries (Torella et al. 2004). While, Urano et al. demonstrated in VSMCs isolated from uninjured or balloon-injured rat aortas that although injury increased VSMC outgrowth and cell number in young vessels, no induction of cell growth following injury was seen in explants from old aortas (Urano et al. 1999). Thus overall, investigations into the effect of age in rodent injury models have produced conflicting results. In patients, however, evidence suggests that ageing may inhibit neointima formation. Goldman and colleagues reported that the amount of time before graft occlusion following coronary artery bypass grafting was increased in older patients (Goldman et al. 2004) and Hugl and co-workers detected less restenosis in carotid endarterectomy patients over 70 years old (Hugl et al. 2006).

Early evidence suggests that Wnt-mediated regulation of VSMC behaviour is impaired with age. A study by Marchand and colleagues demonstrated that Wnt3a-induced proliferation and subsequent expression of cyclin-D1 were diminished in VSMCs from old rats compared to young controls (Marchand et al. 2011). Thus, we hypothesised that the ability of Wnt4 to induce VSMC proliferation may also be impaired with age. To test this hypothesis the effect of ageing on VSMC proliferation both in vitro and during neointima formation in a carotid artery ligation model was analysed. In addition, as reduced Wnt4 expression with age has been reported in non-vascular tissues (Rauner et al. 2008; Kvell et al. 2010; Winkler et al. 2014), expression of Wnt4 protein within the developing neointima was also examined in young and old mice.

## Materials and methods

### Animals

Housing, care and all procedures involving mice were performed in accordance with the guidelines and regulations of the University of Bristol and the United Kingdom Home Office. The investigation conforms to the Guide for the Care and Use of Laboratory Animals published by the US National Institutes of Health (NIH Publication No. 85–23, revised 1996).

### Isolation and culture of VSMCs

VSMCs were isolated from aortas from young (2 month) and 12 old (18–20 month) C57BL6/J mice purchased from Charles River, using the explant procedure and cultured as described previously (Tsaousi et al. 2011). VSMCs were grown in DMEM supplemented with 10% FBS, 2 mM L-glutamine, 100 units/mL penicillin, 100 μg/mL streptomycin and 8 μg/mL gentamycin (10% FBS/DMEM). VSMCs were used between passages 2–10.

### Proliferation - EdU immunofluorescence

To quantify proliferation in vitro*,* 5-ethynyl-2′-deoxyuridine (EdU) immunofluorescence was performed using the Click-iT EdU Alexa Fluor 488 Imaging Kit (C10337, Invitrogen, Paisley, UK) according to the manufacturer’s instructions. VSMCs were seeded onto glass coverslips at 2-4 × 10^4^ cells/well in 24-well plates, allowed to adhere in 10% FBS/DMEM at 37 °C, 5% CO_2_ overnight, and then quiesced for 24–72 h prior to treatment with 10 μM EdU and 10% FBS/DMEM or 400 ng/mL recombinant Wnt4 protein (R&D Systems, 475-WN). VSMCs were incubated for 24 h at 37 °C, 5% CO_2_, then fixed in 3% (*w*/*v*) paraformaldehyde/PBS for 15 min at room temperature. VSMCs were then washed twice in 3% (*w*/*v*) BSA/PBS and permeabilised by incubation in 0.5% (*v*/v) triton/PBS for 20 min at room temperature. Then VSMCs were washed twice in 3% (*w*/*v*) BSA/PBS and incubated with Click-iT reaction cocktail for 30 min at room temperature. VSMCs were washed once in 3% (*w*/*v*) BSA/PBS and then once in PBS. Nuclei were then stained by 30 min incubation with 5 μg/mL Hoechst-33,342 in PBS at room temperature. Coverslips were washed twice in PBS and mounted in polyvinylpyrrolidone solution. EdU positive cells (green nuclei) and negative cells (blue only) were counted in twenty high magnification fields (×600), and the number of EdU positive cells was expressed as a percentage of the total number of cells counted.

### Western blotting

In vitro proliferation was also investigated by analysis of proliferating cell nuclear antigen (PCNA) protein levels using Western blotting. VSMCs were seeded at 8 × 10^4^ cells/well into 12-well plates pre-coated with 10 μg/mL fibronectin (F1141, Sigma Aldrich, Dorset, UK) for two hours at room temperature, and allowed to adhere in 10% FBS/DMEM at 37 °C, 5% CO_2_ overnight. Cells were then quiesced for 24 h in SFM prior to treatment with either SFM or 10% FBS/DMEM for 24 h. Cells were lysed in 5% SDS lysis buffer and protein concentration was measured using the Micro Bicinchoninic Acid Protein Assay Kit (23,235, Thermo Fisher Scientific, Massachusetts, USA). Western blots were performed as previously described (Uglow et al. 2003) using 1 μg/mL PCNA antibody (ab18197, Abcam, Cambridge, UK) diluted in 5% (*w*/*v*) BSA/TBS overnight at 4 °C. Levels of PCNA (optical density (O.D.) x mm^2^) were normalised to the corresponding stain-free band (456–1084, Bio-Rad, Hertfordshire, UK).

### Murine carotid artery ligation

To investigate whether ageing affected intimal thickening and VSMC proliferation in vivo*,* carotid artery ligation was performed on the left common carotid artery of 12 young (2 month) and 12 old (18–20 month) C57BL6/J male mice, as previously described (Tsaousi et al. 2011). Briefly, mice were anesthetized by inhalation of 3% isofluorane in 100% oxygen; the left common carotid artery was located and ligated using a 5–0 silk suture just proximal to the bifurcation. Mice were also given 1.5 μg buprenorphine hydrochloride for analgesia (I.P.). After allowing 21 days for neointima formation to occur, mice were culled using 20 mg pentobarbital sodium (I.P.) and the ligated carotid arteries were dissected and fixed in 10% (*v*/v) formalin/PBS for 24 h. Arteries were then transferred into PBS and stored at 4 °C until processing. In addition to the above, sham operations were performed in young and old mice. As further controls, for each age group four additional mice were employed as unligated controls. These control mice were not subject to carotid ligation and were instead culled using 20 mg pentobarbital sodium at day 0. The left carotid arteries were dissected, fixed and stored as described above.

### Histological processing and staining

Blood vessels were embedded in agar plugs then processed and embedded in paraffin wax. Then 3 μm transverse sections were cut and mounted onto Superfrost Plus slides. To analyse vessel structure Elastin van Gieson (EVG) staining was performed and analysed using ImageJ software. To quantify neointimal cell number and density, sections were stained with 4′,6-diamidino-2-phenylindole (DAPI), the intimal cell number was counted and normalised to neointimal area. Alternatively, to analyse proliferation, colorimetric immunohistochemistry for proliferating cell nuclear antigen (PCNA) was performed (1 μg/mL, ab18197, Abcam, Cambridge, UK) with 3,3′-diaminobenzidine (DAB) and the number of PCNA positive nuclei (brown) were counted in four ×60 images and expressed as a percentage of the total number of cells. To investigate Wnt4 protein expression, immunofluorescence was performed (5 μg/mL, sc13692, Santa Cruz Biotechnology, Heidelberg, Germany) and Wnt4 content in the neointima was measured by pixel analysis using the Image-Pro Software. Pixel counts were normalized to neointimal area to generate the percentage of neointimal area with positive pixels. In both cases, the same concentration of non-immune IgG from the corresponding species was employed as a negative control to confirm primary antibody specificity. To identify apoptotic cells in situ DNA end labelling (ISEL) was performed. Firstly, sections were digested with 5 μg/mL of proteinase-K diluted in Tris/EDTA buffer (10 mM Tris.Cl, 1 mM Ethylenediaminetetraacetic acid (EDTA)) for 15 min at room temperature before ISEL was performed as previously described (George et al. 2001). The number of ISEL positive nuclei (brown) were counted in four ×60 images and expressed as a percentage of the total number of cells.

### Statistics

Statistical analysis was performed using Graphpad Instat statistical software. Normal distribution of data was assessed using a Kolmogorov and Smirnov test for normality. Means of two groups were compared using a Student’s t-test, t-test with Welch Correction or a Mann Whitney test, as appropriate depending on whether standard deviations were equal. Paired or unpaired analysis was used as appropriate. An output of *p* < 0.05 was accepted as significantly different in all statistical tests. All graphical data was expressed as mean ± standard error of the mean (SEM).

## Results

### Wnt4-induced proliferation was impaired in VSMCs from old mice

**Fig. 1:**
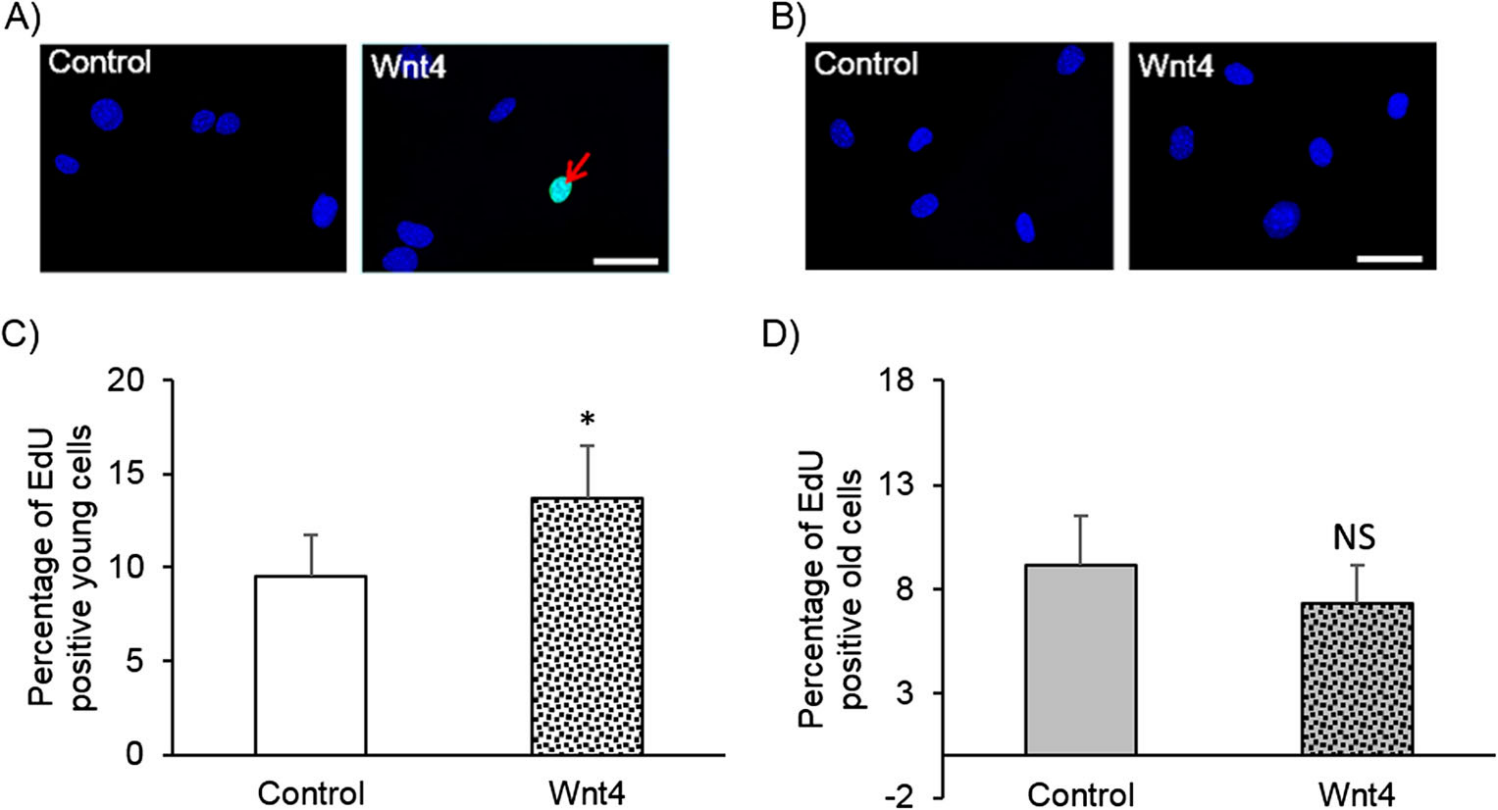
Wnt4 protein induced proliferation in VSMCs from young but not old mice. Proliferation was quantified in aortic VSMCs isolated from young and old mice and stimulated with 400 ng/mL recombinant Wnt4 protein for 24 h using immunofluorescence detection of EdU incorporation. Representative images are shown (**a** and **b** for young and old VSMCs, respectively). The red arrow indicates a positive cell. The scale bar represents 25 μm and applies to all images. The number of EdU positive cells (green) was counted and expressed as a percentage of the total number of cells (blue nuclei: hoechst) (**c**, **d**). * indicates *p* < 0.05 vs. control, NS indicates not significant, paired Student’s t-test_,_ *N* = 6

Firstly, the effect of age on VSMC proliferation in vitro was assessed in serum-free conditions or 10% serum (FBS). Supplementary figure 1 shows that FBS-induced proliferation, as measured by EdU incorporation by immunocytochemistry or PCNA quantification by Western blotting, did not significantly differ between VSMCs from young and old mice. Thereafter the effect of age on Wnt4-induced VSMC proliferation was investigated. Figure 1 shows that although recombinant Wnt4 protein significantly increased proliferation in VSMCs from young mice, this response was absent in VSMCs from old mice. Together these data suggest that although basal and FBS-induced proliferation were unaffected by ageing, the ability of Wnt4 to induce VSMC proliferation was lost with age.

### Carotid artery ligation induced intimal thickening was unaffected by age

To investigate the effect of age on intimal thickening, ligation of the left carotid artery was performed in young and old mice. 21 days after surgery arteries were removed, processed for histology, sectioned transversely and EVG staining was performed. To analyse neointima length, transverse sections were chosen at three points along the left carotid artery; immediately adjacent to the ligature, 100 μm and 200 μm distal from the ligature. Immediately adjacent to the ligature, neointima formation was observed in all vessels. At 100 μm distal to the ligature, 10 young and 9 old vessels retained a visible neointima, while at 200 μm distal to the ligature, neointimas were observed in 6 young and 5 old vessels. Thus at all sites investigated, the number of vessels containing a neointima was similar in old and young mice, suggesting that neointimal length after carotid artery ligation was not affected by ageing.

**Fig. 2:**
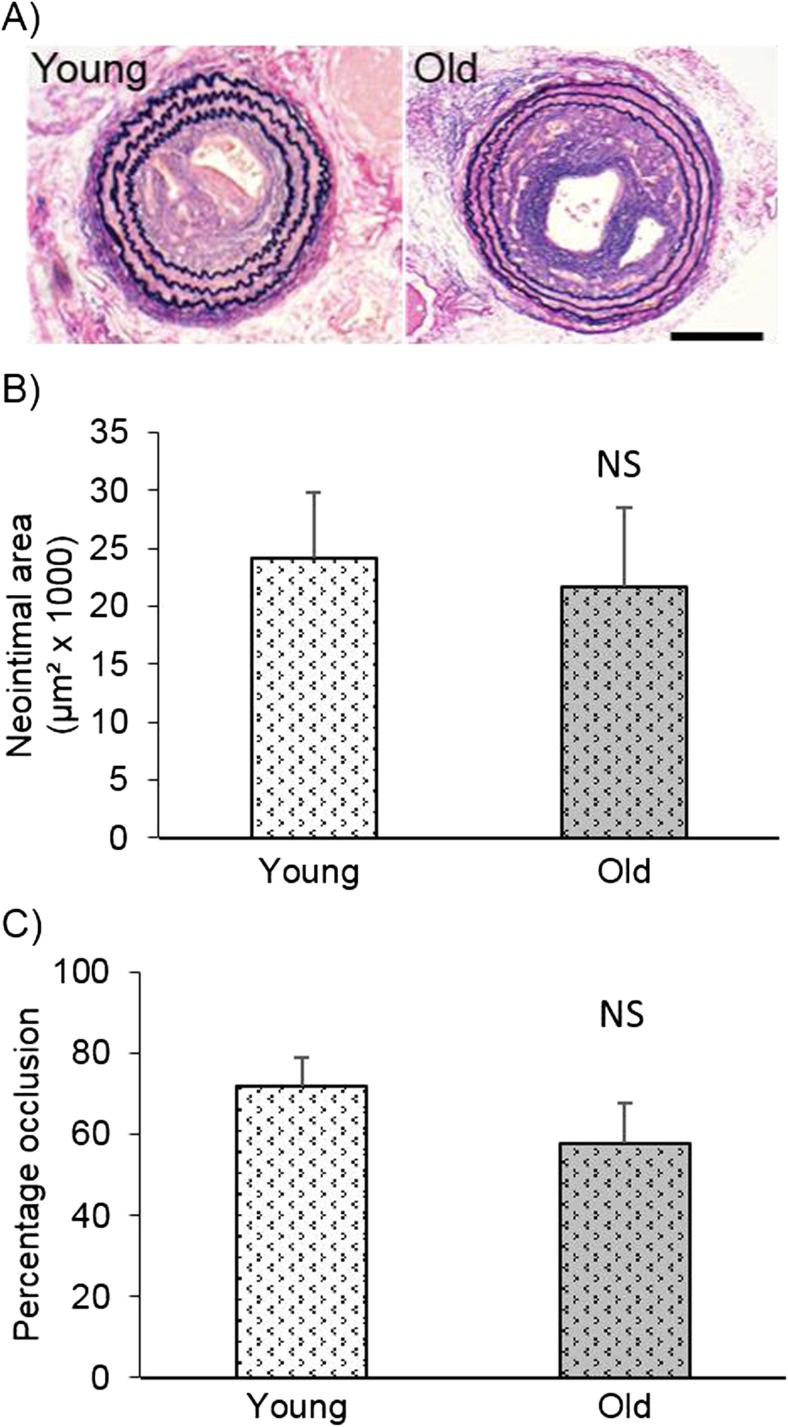
Neointimal area and percentage occlusion were unaffected by age. Left carotid arteries were taken from young and old mice 21 days after ligation and transverse sections were taken immediately adjacent to the ligature. Representative images of EVG-stained left carotid arteries are shown (**a**) and the neointimal area (**b**) and percentage occlusion of the lumen (**c**) were analysed. No significant differences (NS) were observed with age, unpaired Student’s t-test, *N* = 12. The scale bar represents 150 μm and applies to both images

Neointimal size and percentage occlusion was also measured at these three sites; data from immediately adjacent to the ligature are shown in Fig. 2, while data from 100 μm and 200 μm distal from the ligature are shown in supplementary figure 2. As expected, the size of the lesion was largest near the ligation and smallest at 200 μm from the ligature. At all three sites investigated, neointimal size and percentage occlusion did not significantly differ with age. As a similar conclusion was determined from analysis at each of the three sites, further immunohistochemistry on these vessels was performed on sections taken adjacent to the ligature.

Importantly, no thickening of the intima was observed in control arteries from young or old mice which had not undergone the ligation procedure (supplementary figure 3). This demonstrates that the thickening intima observed in ligated vessels was due to the ligation procedure itself and not due to natural ageing of the vasculature. This lack of neointima in uninjured carotid arteries from young and aged mice has also been described previously (Vazquez-Padron et al. 2004). Furthermore, in the current study we did not observe any neointima formation in mice subjected to a sham operation (data not shown).

### Neointimal Wnt4 protein expression was unaffected by age

**Fig. 3:**
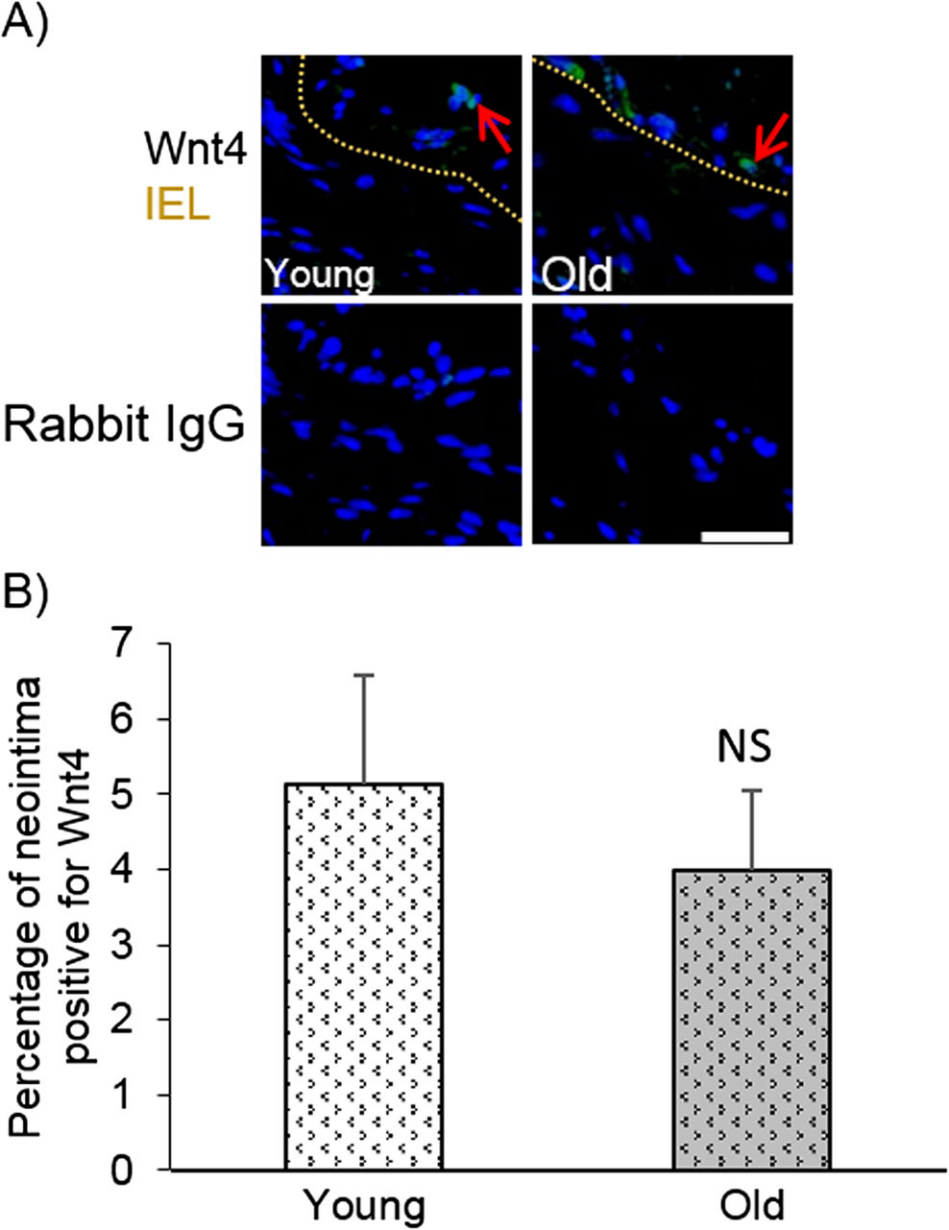
Neointimal Wnt4 protein expression was unaffected by age. Left carotid arteries were taken from young and old mice 21 days after ligation and immunohistochemistry for Wnt4 was performed on transverse sections taken adjacent to the ligature. **a** Representative images of Wnt4 immunofluorescence (green) with DAPI stained nuclei (blue). The top row shows representative images including a yellow dashed line indicating the media-intima boundary (internal elastic lamina, IEL) plus red arrows pointing to examples of Wnt4 positive cells. The bottom row shows corresponding non-immune rabbit IgG negative control images. The scale bar represents 50 μm and applies to all images. **b** Wnt4 positive pixels (green) in the neointima of left carotid arteries were quantified and expressed as a percentage of the neointimal area. No significant difference (NS) was observed with age, unpaired Student’s t-test, *N* = 12

To determine whether neointimal expression of Wnt4 was affected by age, left carotid arteries were stained for Wnt4 protein and the positive intimal area was quantified by pixel analysis. The percentage of the neointima positive for Wnt4 protein did not significantly differ with age (Fig. 3).

### Neointimal cell number, proliferation and apoptosis were unaffected by age

**Fig. 4:**
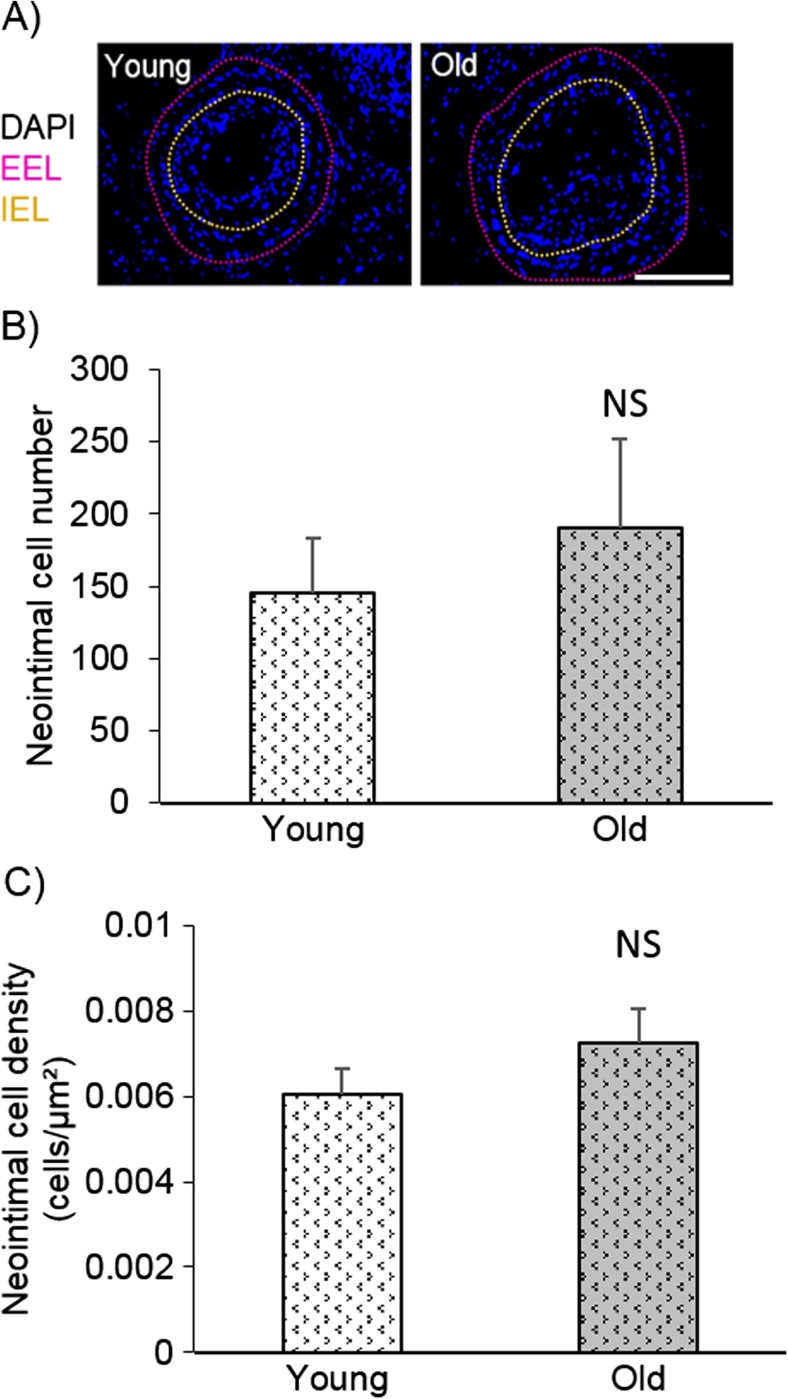
Neointimal cell number and density were unaffected by age. Left carotid arteries were taken from young and old mice 21 days after ligation and DAPI staining was performed on transverse sections taken adjacent to the ligature. Representative images with DAPI stained nuclei (blue) including a purple dashed line indicating the media-adventitia boundary (external elastic lamina, EEL) and a yellow dashed line indicating the media-intima boundary (internal elastic lamina, IEL) (**a**). The scale bar represents 150 μm and applies to all images. Neointimal cell counts were performed (**b**) and cell density was calculated by normalising the cell number to neointimal area (**c**). No significant differences (NS) were observed with age, unpaired Student’s t-test, *N* = 12 young and *N* = 11 old

To determine whether the amount of cells populating the neointima was affected by age, the number of intimal nuclei were counted in DAPI stained sections (Fig. 4a). Furthermore, this cell number was normalised to neointimal area to calculate cell density. Neither the neointimal cell number (Fig. 4b) nor density (Fig. 4c) were affected by age.

**Fig. 5:**
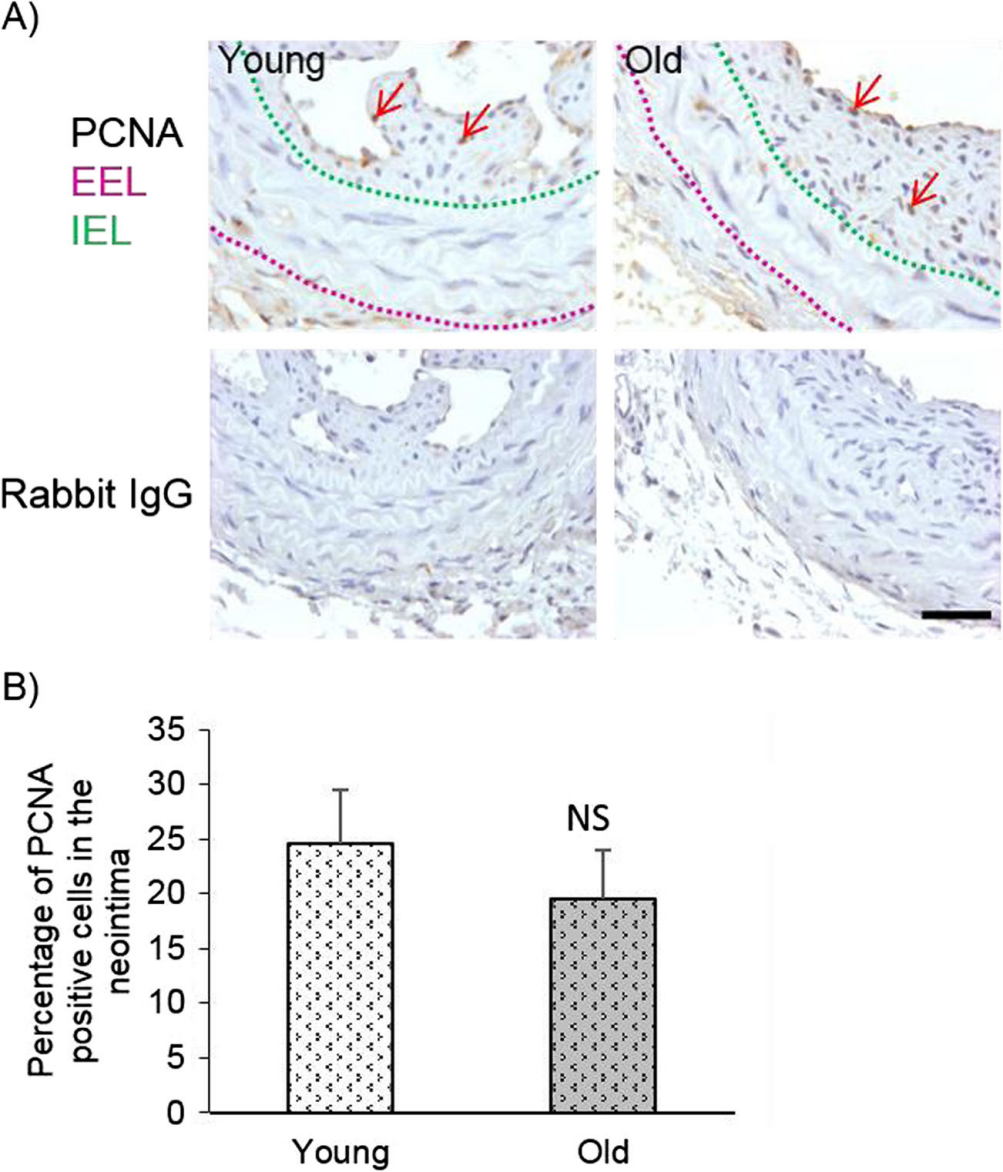
Neointimal cell proliferation was unaffected by age. Proliferation was quantified in left carotid arteries from young and old mice 21 days after ligation by proliferating cell nuclear antigen (PCNA) immunohistochemistry on transverse sections taken adjacent to the ligature. **a** Representative images are shown including a purple dashed line indicating the media-adventitia boundary (external elastic lamina, EEL), and a green dashed line indicating the media-intima boundary (internal elastic lamina, IEL) plus red arrows pointing to examples of PCNA positive nuclei. The bottom row shows the corresponding non-immune rabbit IgG negative control images. The scale bar represents 50 μm and applies to all images. **b** The proportion of nuclei in the intima staining positive for PCNA (brown nuclei) was expressed as a percentage of the total number of intimal nuclei viewed (blue nuclei: hematoxylin). No significant difference (NS) was observed with age, unpaired Student’s t-test, N = 12

**Fig. 6:**
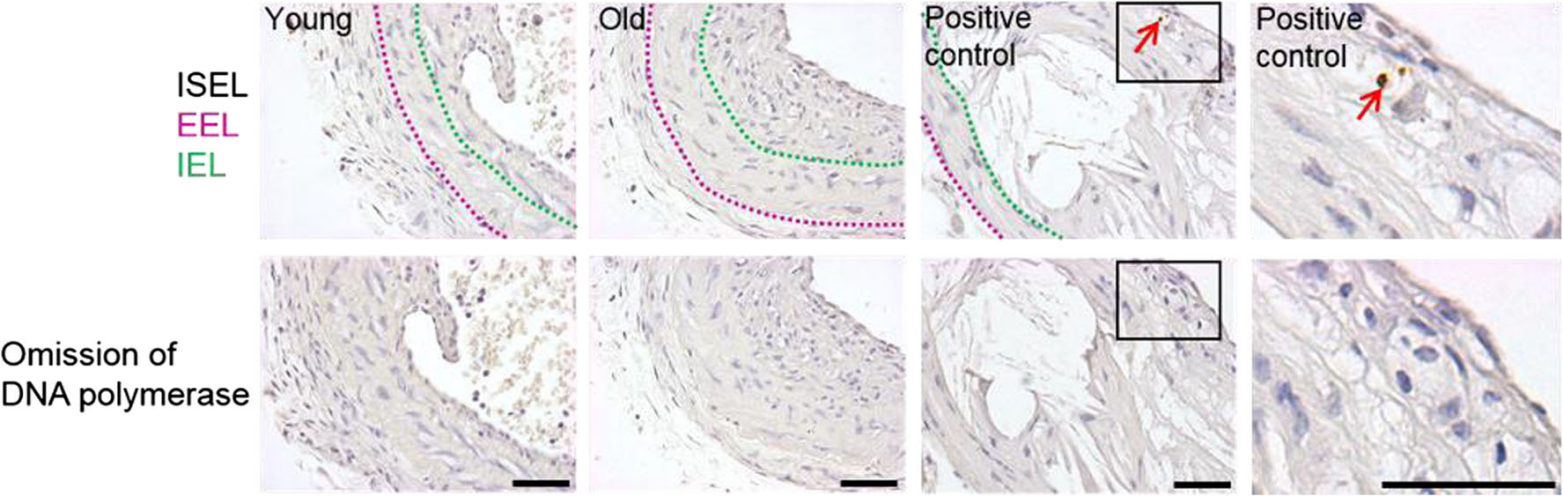
Neointimal cell apoptosis was negligible and unaffected by age. Apoptosis was assessed by in situ DNA end labelling (ISEL) in left carotid arteries from young and old mice 21 days after ligation. ISEL staining was performed on transverse sections taken adjacent to the ligature (*N* = 5). Negligible levels of ISEL positive cells were observed. Representative images of ISEL-stained ligated left carotid arteries are shown along with the positive control of a murine brachiocephalic atherosclerotic plaque from a male apolipoprotein-E deficient mouse fed high fat diet for 12 weeks (protocol previously described by Johnson et al. 2005). ISEL positive cells have brown shrunken nuclei, negative cells have blue nuclei (hematoxylin). The top row shows representative images including a purple dashed line indicating the media-adventitia boundary (external elastic lamina, EEL), and a green dashed line indicating the media-intima boundary (internal elastic lamina, IEL) plus red arrows pointing to an ISEL-positive nuclei in the positive control. The bottom row shows the corresponding negative control images (omission of DNA polymerase). The scale bar represents 50 μm and applies to the all images in the above column

The effect of age on neointimal proliferation and apoptosis was then investigated by PCNA immunohistochemistry and ISEL, respectively. The proportion of proliferative and apoptotic cells in the neointima was similar in arteries from old and young mice (Figs. 5 and 6, respectively). Together these data suggest that neither neointimal proliferation, apoptosis nor resultant cell number after carotid artery ligation were altered with ageing.

## Discussion

In vitro experiments in this paper demonstrated that basal and FBS-induced VSMC proliferation were unaffected by age. This finding contrasts other reports of ageing mouse VSMC proliferation within the literature. For instance, Moon and colleagues reported significantly reduced proliferation in aortic VSMCs isolated from old mice under quiesced control conditions or when stimulated with FBS, α-thrombin and the lipid peroxidation product 4-hydroxynonenal (Moon et al. 2001). Similarly, Rodriguez-Menocal and co-workers reported that with age, a larger percentage of mouse VSMCs entered growth arrest after serum starvation in culture (Rodriguez-Menocal et al. 2010). In a separate study by Vazquez-Padron et al. although no difference in proliferation was observed when old and young mouse VSMCs were incubated in 2% FBS alone, a significantly faster growth rate with age was reported in VSMCs stimulated simultaneously with 2% FBS and platelet-derived growth factor (PDGF)-BB (Vazquez-Padron et al. 2004).

In contrast, we found that the ability of recombinant Wnt4 protein to induce VSMC proliferation was lost with age. This is the first report of impaired Wnt4 signalling in VSMCs with ageing. However, this finding is supported by a previous study by Marchand and colleagues describing reduced Wnt3a-mediated proliferation in VSMCs from aged rats. Interestingly, Marchand et al. also found that serum-induced proliferation was unaffected by age (Marchand et al. 2011), thus it is tempting to speculate that these results imply a specific impairment in the Wnt signalling pathway in VSMCs from aged rodents.

Initially, it was hypothesised that loss of Wnt4-induced proliferation with age in vivo would result in reduced neointima formation, similar to that previously reported in Wnt4 heterozygous mice (Tsaousi et al. 2011). However, we observed that following carotid artery ligation, the number of arteries containing a neointima, the neointima area and the percentage occlusion recorded at three points distal to the ligature did not differ with age. In addition, neither neointimal cell number, density, proliferation nor apoptosis were altered in old arteries compared to young controls. Together these findings suggest that, in contrast to our hypothesis, although Wnt4-mediated proliferation was lost with age in vitro*,* no difference in proliferation was observed in vivo*.* This may suggest that VSMCs within the neointima in vivo retain their sensitivity to Wnt4 despite increasing age. It is possible that insensitivity of VSMCs from old mice to Wnt4 may be an artefact of culture and passaging in vitro. It has previously been suggested that removal of VSMCs from the mechanical and chemical signals present in the arterial wall may have differential effects with age (Bochaton-Piallat et al. 1993). Bochaton-Piallat and colleagues reported that in culture a greater percentage of VSMCs isolated from old rats lost their in vivo phenotype, measured by α-smooth muscle-actin expression, compared to VSMCs isolated from young adult or new-born counterparts. The authors hypothesised that old VSMCs may require environmental signals from the artery to maintain contractile protein expression, whereas in young cells continued expression of α-smooth muscle-actin was intrinsic (Bochaton-Piallat et al. 1993). It is possible that in culture VSMCs from old mice lose their ability to respond to Wnt4, whereas young cells maintain sensitivity to this Wnt despite removal from the arterial environment. Alternatively, it is possible that sensitivity to Wnt4 was in fact lost with age in vivo*,* but compensatory upregulation of expression or sensitivity to another mitogens occurred, thus accounting for the similar overall cell proliferation and intimal area observed in young and old vessels. For instance, Vazquez-Padron and colleagues reported increased expression of PDGF receptor-α with age in the uninjured mouse aorta and increased PDGF-BB induced growth of primary VSMCs isolated from old mice compared to young controls (Vazquez-Padron et al. 2004). It is possible that a greater role of other mitogens, such as PDGF, with age may counteract any reduced sensitivity to Wnt4 in aged vessels.

To our knowledge, this is the first investigation of the effect of age on neointima formation and proliferation using the carotid artery ligation model in mice. Interestingly, a study employing a wire induced injury model in the mouse carotid artery has reported increased neointima formation and cell density accompanied by reduced neointimal cell apoptosis with age, in complete contrast to the results described here (Vazquez-Padron et al. 2004). This discrepancy may be due to the differing techniques used to induce intimal thickening. While ligation induced neointima formation involves blood stasis and formation of a fibrin scaffold for VSMC migration (Kumar and Lindner 1997; Kawasaki et al. 2001), wire induced carotid injury entails endothelial denudation and platelet adhesion to the intima (Lindner et al. 1993; Vazquez-Padron et al. 2004). It is possible that cellular or molecular mechanisms involved in wire induced, but not ligation induced, intimal thickening may be affected by age thus explaining the divergent results between these two studies. This would not be an unfounded suggestion as Choi and colleagues previously reported that the effect of β_3_ integrin knockout on neointimal thickening differed depending on whether injury was induced using a guidewire probe or ligation (Choi et al. 2004). Hence it could be proposed that the cellular mechanisms involved in wire induced neointima formation, such as endothelial repair, are affected by age whilst those contributing to both wire and ligation induced intimal thickening, such as VSMC proliferation and migration, are not. Further investigation would be necessary to confirm this.

Wnt4 expression is reported to be reduced with age in multiple non-vascular tissues including murine bone (Rauner et al. 2008), intervertebral disc (Winkler et al. 2014), brain (Hofmann et al. 2014) and thymic epithelia (Kvell et al. 2010). It was therefore hypothesised that neointimal Wnt4 expression may also be reduced with age. However, the percentage of the neointima staining positive for Wnt4 protein did not significantly differ between arteries from young and old mice.

This study reveals that although Wnt4-induced proliferation was lost with age in primary VSMCs*,* no difference in neointimal formation, cell density or proliferation was observed with age in a model of carotid artery ligation. These results may imply that Wnt4-mediated proliferation is unaffected by age in vivo*,* suggesting that inhibition of the Wnt4 signalling pathway may represent a therapeutic target to inhibit restenosis in patients of all ages. The need for therapies to treat restenosis in elderly patients will become an increasing problem in the current ageing population, as patients would be expected live for many decades following balloon angioplasty, stenting or coronary artery bypass surgery.

## Electronic supplementary material

Supplementary Figure 1 Basal and serum-induced VSMC proliferation were unaffected by age. A-B) Proliferation was quantified in aortic VSMCs isolated from young and old mice and treated with serum-free medium (SFM) or 10% (*v*/v) FBS/DMEM (FBS) for 24 h using immunofluorescence detection of EdU incorporation. A) Representative images are shown, red arrows indicate positive cells. The scale bar represents 25 μm and applies to all images. B) The number of EdU positive cells (green) was counted and expressed as a percentage of the total number of cells viewed (blue nuclei: hoechst). No significant differences (NS) were observed between young and old VSMCs under either condition. An unpaired t-test with Welch correction was used for SFM data while an unpaired Student’s t-test was used for FBS data_,_ *N* = 6 young and *N* = 5 old. C-F) Proliferating cell nuclear antigen (PCNA) protein was detected by Western blotting of aortic VSMCs isolated from young and old mice and treated with serum-free medium (SFM) or 10% (*v*/v) FBS/DMEM (FBS) for 24 h. Representative Western blots are shown for SFM (C) and FBS (D). Levels of PCNA protein in VSMCs treated with SFM (E) or FBS (F) for 24 h were normalised to the corresponding stain-free band. No significant differences (NS) were observed between young and old VSMCs under either condition, unpaired Student’s t-test, *N* = 3. (DOCX 258 kb)

Supplementary Figure 2 Neointimal area and percentage occlusion at 100 μm and 200 μm distal to the ligature were unaffected by age. The neointimal area and percentage occlusion of the lumen were analysed in left carotid arteries from young and old mice 21 days after ligation. Transverse sections were taken 100 μm and 200 μm distal to the ligature and measurements of EVG stained vessel parameters were performed. Representative images are shown for 100 μm (A) and 200 μm (D). The scale bar represents 150 μm and applies to both images. Neointimal area and percentage occlusion were analysed at 100 μm (B & C) and 200 μm (E & F). No significant differences (NS) were observed with age, Mann Whitney tests were employed to analyse neointimal area, whereas unpaired Student’s t-test were used for percentage occlusion, *N* = 12. (DOCX 571 kb)

Supplementary Figure 3 Representative images of unligated left carotid arteries from young and old mice. Representative images of EVG-stained unligated left carotid arteries from young and old mice. Scale bar represents 150 μm and applies to both images. (DOCX 225 kb)

## Abbreviations

DAB: 3,3′-diaminobenzidine
EDTA: Ethylenediaminetetraacetic acid
EdU: 5-ethynyl-2′-deoxyuridine
EEL: External elastic lamina
EVG: Elastin van Gieson
IEL: Internal elastic lamina
ISEL: In situ DNA end labelling
PCNA: Proliferating cell nuclear antigen
PDGF: Platelet-derived growth factor
SFM: Serum-free medium
TCF: T-cell factor
VSMC: Vascular smooth muscle cell
Wnt: Wingless/Int
