# Supplementary figures and images for: Carotid artery ligation induced intimal thickening and proliferation is unaffected by ageing
Source: J Cell Commun Signal. 2017 Nov 29;12(3):529–37. doi: 10.1007/s12079-017-0431-5 (PMC6039339; doi:10.1007/s12079-017-0431-5)

Supplementary Figure 1:


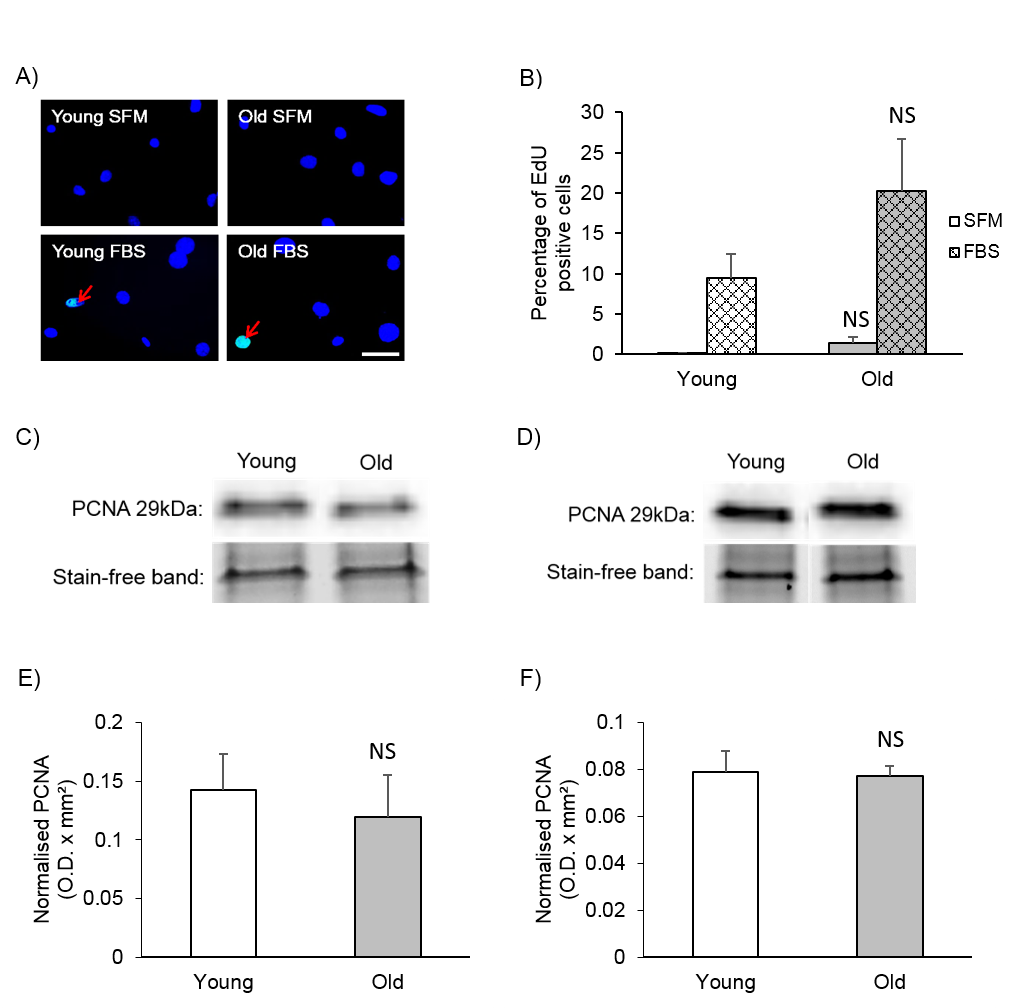

Supplement: Supplementary file 1 — Basal and serum-induced VSMC proliferation were unaffected by age. A-B) Proliferation was quantified in aortic VSMCs isolated from young and old mice and treated with serum-free medium (SFM) or 10% (v/v) FBS/DMEM (FBS) for 24 h using immunofluorescence detection of EdU incorporation. A) Representative images are shown, red arrows indicate positive cells. The scale bar represents 25 μm and applies to all images. B) The number of EdU positive cells (green) was counted and expressed as a percentage of the total number of cells viewed (blue nuclei: hoechst). No significant differences (NS) were observed between young and old VSMCs under either condition. An unpaired t-test with Welch correction was used for SFM data while an unpaired Student’s t-test was used for FBS data, N = 6 young and N = 5 old. C-F) Proliferating cell nuclear antigen (PCNA) protein was detected by Western blotting of aortic VSMCs isolated from young and old mice and treated with serum-free medium (SFM) or 10% (v/v) FBS/DMEM (FBS) for 24 h. Representative Western blots are shown for SFM (C) and FBS (D). Levels of PCNA protein in VSMCs treated with SFM (E) or FBS (F) for 24 h were normalised to the corresponding stain-free band. No significant differences (NS) were observed between young and old VSMCs under either condition, unpaired Student’s t-test, N = 3. (DOCX 258 kb) [file 12079_2017_431_MOESM1_ESM.docx]

Supplementary Figure 2:


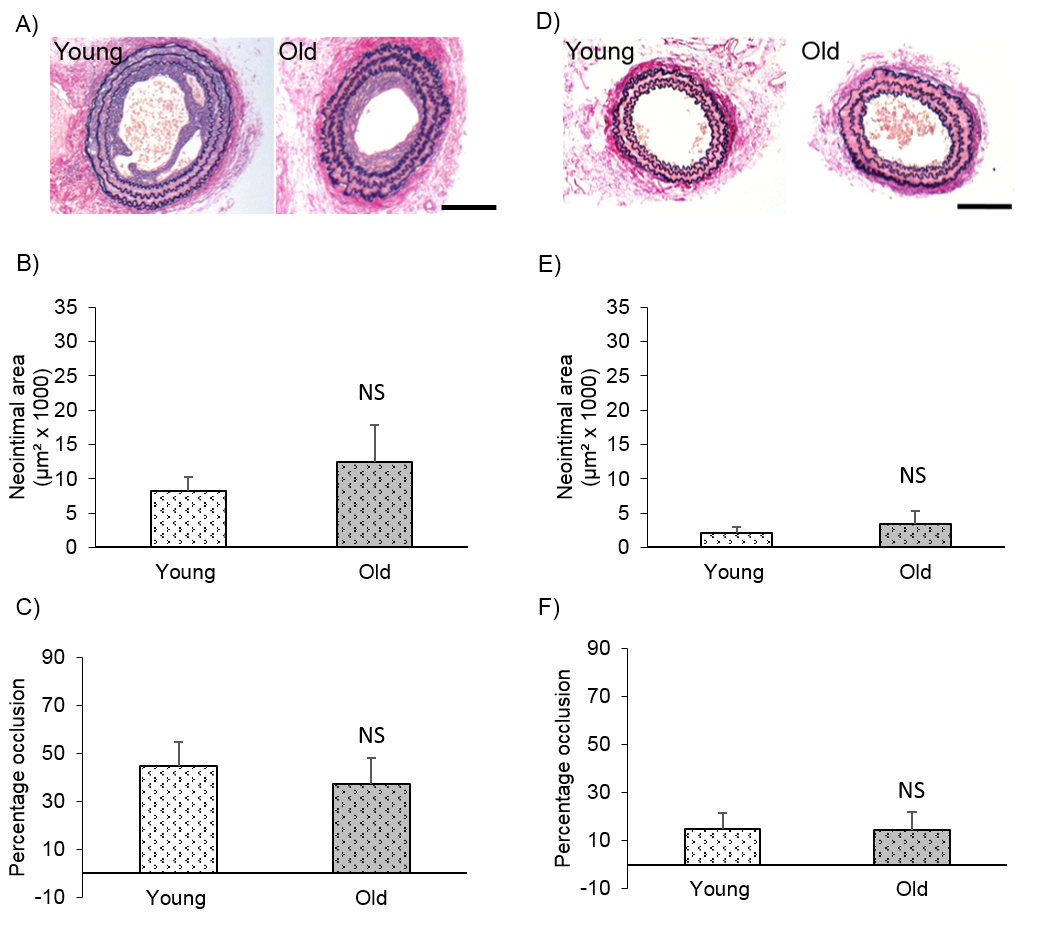

Supplement: Supplementary file 2 — Neointimal area and percentage occlusion at 100 μm and 200 μm distal to the ligature were unaffected by age. The neointimal area and percentage occlusion of the lumen were analysed in left carotid arteries from young and old mice 21 days after ligation. Transverse sections were taken 100 μm and 200 μm distal to the ligature and measurements of EVG stained vessel parameters were performed. Representative images are shown for 100 μm (A) and 200 μm (D). The scale bar represents 150 μm and applies to both images. Neointimal area and percentage occlusion were analysed at 100 μm (B & C) and 200 μm (E & F). No significant differences (NS) were observed with age, Mann Whitney tests were employed to analyse neointimal area, whereas unpaired Student’s t-test were used for percentage occlusion, N = 12. (DOCX 571 kb) [file 12079_2017_431_MOESM2_ESM.docx]

Supplementary Figure 3:


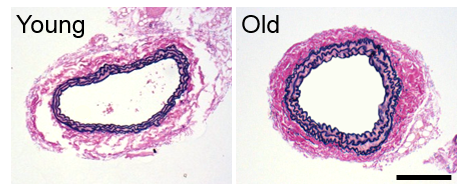

Supplement: Supplementary file 3 — Representative images of unligated left carotid arteries from young and old mice. Representative images of EVG-stained unligated left carotid arteries from young and old mice. Scale bar represents 150 μm and applies to both images. (DOCX 225 kb) [file 12079_2017_431_MOESM3_ESM.docx]
